# Action prediction modulates self–other integration in joint action

**DOI:** 10.1007/s00426-022-01674-y

**Published:** 2022-05-04

**Authors:** Anouk van der Weiden, Emanuele Porcu, Roman Liepelt

**Affiliations:** 1 Institute of Psychology, Leiden University, PO Box 9555, 2300 RB Leiden, The Netherlands; 2 Department of Biological Psychology, Otto-Von-Guericke-University, Magdeburg, Germany; 3 Department of General Psychology: Judgment, Decision Making, Action, Faculty of Psychology, FernUniversität in Hagen, Hagen, Germany

## Abstract

People often coordinate actions with others, requiring an adjustable amount of self–other integration between actor’s and co-actor’s actions. Previous research suggests that such self–other integration (indexed by the joint Simon effect) is enhanced by agent similarity of the co-actor (e.g., high in intentionality). In this study, we aimed to extend this line of research by testing whether experiencing agency over a co-actor’s actions (vicarious agency) and/or action prediction strengthens the joint Simon effect. For this purpose, we manipulated experienced agency by varying the experienced control over a co-actor’s actions (Experiment 1), and action prediction regarding the co-actor’s actions (Experiment 2). Vicarious agency could effectively be induced, but did not modulate the size of the joint Simon effect. The joint Simon effect was decreased when the co-actor’s actions were unpredictable (vs. predictable) during joint task performance. These findings suggest social agency can be induced and effectively measured in joint action. Action prediction can act as an effective agency cue modulating the amount of self–other integration in joint action.

## Introduction

One characteristic of the human species is that we evolved in social coevolution with others. We often perform actions together with or in the proximity of others, such as avoiding to bump into others at a busy train station, moving a table together, or hitting balls on only one side of the tennis court when playing doubles. Such action coordination requires us to represent and anticipate our own as well as others’ actions and action-effects, also referred to as action co-representation (Gabbard & Bobbio, 2011; Oliveira & Ivry, 2008; Pezzulo, 2011). Although the representation of other people’s actions seems to occur quite naturally (Carr et al., 2003; Cochin et al., 1999; Hari et al., 1998; Mukamel et al., 2010), action co-representation and joint-action coordination are not as simple as it seems. Sometimes people fail to represent another person’s actions correctly, or represent another person’s actions as if they were their own. Both can result in an inability to (re)act adequately. For example, when two piano players perform different parts of a piano duet, failing to represent the other person’s actions correctly would make it impossible to time one’s own actions accordingly, while representing the other person’s actions as if they were one’s own would result in the urge to actually play the other person’s part as well. This raises the intriguing question how people represent the actions of others, and whether and how this representation differs from the representation of their own actions?

A famous paradigm to test the question of how people represent the actions of others is the social or joint Simon paradigm (Dolk et al., 2013; Sebanz et al., 2003). In this paradigm, the classic Simon task (Craft & Simon, 1970; Simon et al., 1970; Simon & Small Jr., 1969) is distributed (shared) between two persons. Each person sitting side-by-side is responsible for one of two different items of a specific category (e.g., form or color), responding by pushing one of two laterally located response keys. The stimuli are laterally presented on the left or right side of a computer screen. Even though stimulus location is task-irrelevant for both persons, studies typically show a Stimulus–Response (S–R) compatibility effect (Liepelt et al., 2011; Sebanz et al., 2003)—faster reaction times when stimulus and response location correspond (S–R compatible), compared to when they do not correspond (S–R incompatible), similar to the classical Simon task where one person takes care of both responses (Craft & Simon, 1970; Simon & Wolf, 1963). This joint Simon effect typically fully breaks down when one person of the couple sharing the task performs their half of the joint Simon task without a co-actor responding to the alternative stimulus (Liepelt & Prinz, 2011; Sebanz et al., 2003). This result indicates that the longer reaction times on incompatible trials in the joint version of the Simon task emerge because participants co-represent their co-actor’s actions.

Based on the assumption of functional similarity between the standard Simon effect (Simon & Small Jr., 1969) and the joint Simon effect, the other person’s action is regarded similar to one’s own action (Sebanz et al., 2003) and the other’s task is regarded similar to one’s own task (Sebanz et al., 2005). As such, the joint Simon effect can be explained by an automatic action co-representation mechanism (Sebanz et al., 2003, 2005).

When coordinating their own actions in individual task settings, it has been shown that people need to discriminate their responses through referencing their action to a particular spatial response code (Ansorge & Wühr, 2004) termed ‘referential coding’ (Elsner & Hommel, 2001; Hommel, 1993, 1996; Simon, 1990). For example, when typing on a keyboard, one may represent left-hand button presses as ‘left’ and right-hand button presses as ‘right’. Similarly, when sharing the Simon task with another person, the joint Simon effect has been shown to be based on the need to discriminate the responses of both persons sharing the task (i.e. self–other discrimination) through a specific spatial response code (Dolk et al., 2011, 2013; Klempova & Liepelt, 2016; Liepelt et al., 2016). This has also been termed referential coding, the coding of the actor’s action in reference to their interaction partner’s action (e.g., as left versus right; Dolk et al., 2013, 2014; Sebanz et al., 2003; Tsai et al., 2008). Independent of the specific account proposed for the joint Simon effect, most accounts do agree that it represents a valid measure of self–other integration (Colzato et al., 2012; Dolk et al., 2014; Iani et al., 2011).

The integration of one’s own and others’ actions seems to crucially depend on the extent to which both persons perceive their co-actor’s actions to be similar to their own. That is, action integration is typically stronger when people have to coordinate their own (e.g., left and right hand) actions (i.e., standard Simon effect) compared to when they have to coordinate their actions with the actions of others (i.e., joint Simon effect/self–other integration). Also, people typically show stronger self–other integration when their co-actor (whether they are other people, robots, or objects) is more (like themselves) perceived as intentional (Atmaca et al., 2011; Müller et al., 2015; Müller, Brass, et al., 2011; Müller, Kühn, et al., 2011; Stenzel et al., 2012), high in agency (Stenzel et al., 2014), as belonging to the same group (Aquino et al., 2015; Costantini & Ferri, 2013; McClung et al., 2013; Müller, Brass, et al., 2011; Müller, Kühn, et al., 2011) or being in cooperation (Hommel et al., 2009; Iani et al., 2011, 2014; Liepelt & Raab, 2021). Yet, although there is evidence to suggest that agentic characteristics of the co-actor enhance the extent of self–other integration, to our knowledge there has been no research on the effect of experiencing oneself to be the agent of one’s co-actor’s actions.

The experience of agency appears quite natural to us; we think we know very well whether or not we caused an action or subsequent outcome ourselves. However, we sometimes experience agency over actions or outcomes that we did not cause ourselves (Wegner, 2002). Such vicarious agency has been defined as the misattribution of another’s action to the self (Silver et al., 2021). In a groundbreaking study on vicarious agency, Wegner et al. (2004) let participants watch themselves in a mirror while the hands of a confederate (who was hidden from view) appeared where the participant’s hands would normally appear. The hands performed a series of movements, which were announced to participants (activating a representation of the confederate’s actions) either before or after they observed the different movements. Results showed that participants experienced vicarious agency over another person’s actions when a representation of the movements was pre-activated by means of the auditory announcements.

Since this first demonstration of the effect of vicarious agency (the experience of control over movements of others), many studies have conceptually replicated this finding in the context of various actions and outcomes (e.g., stopping a moving square on a specific location, monitoring one’s own arm movements, or causing other people’s emotions or eye movements) (Aarts et al., 2005; Bolt & Loehr, 2017; Dewey & Carr, 2013; Gentsch & Schütz-Bosbach, 2011; Jones et al., 2008; Linser & Goschke, 2007; Ruys & Aarts, 2012; Stephenson et al., 2018; van der Weiden et al., 2011a; 2016; van der Wel et al., 2012) revealing a key role of prior consistent thoughts about action in determining agency (Wegner et al., 2004), which would allow to predict upcoming actions.

It is important to note, however, that the predictability of action-outcomes does not guarantee the experience of agency. That is, even when an event is highly predictable (e.g., that the elevator will go up when a specific button is pressed) people usually do not experience agency when the event is clearly caused by someone else (e.g., when you were not the one who pressed the button; see also the exclusivity principle in Wegner’s theory of apparent mental causation, Wegner, 2002). In line with this notion, it has been shown that the implicit feeling of agency does not emerge in the absence of any action execution, even when participants were able to fully predict a brief tactile sensation on the index finger (to mimic the sensation of action performance) and a resulting auditory stimulus (Antusch et al., 2021). However, although people may not experience that they caused someone else’s actions and outcomes themselves, action predictability may enhance a sense of joint agency (or we-agency) in the context of joint action coordination. Indeed, the predictability of the actions of one’s co-actor has been shown to enhance the feeling of shared control over joint actions and consequences (Bolt & Loehr, 2017; see also Pacherie, 2012). The question remains, however, whether experiences of agency and/or action predictability affect self–other integration during joint action performance.

The role of action prediction in self–other integration has already been proposed quite some time ago (Sebanz et al., 2006), but to our knowledge the current study is the first to empirically test if action prediction in fact modulates self–other integration in the joint Simon task. Therefore, in the present study, we investigate the role of vicarious agency (Experiment 1) and action prediction (Experiment 2) over another person’s actions for self–other integration in joint action. Based on the notion that a greater agent similarity has been shown to increase the joint Simon effect (Atmaca et al., 2011; Hommel et al., 2009; Stenzel et al., 2014), we predict self–other integration to be stronger when one experiences vicarious agency over the co-actor’s actions, and also when the co-actor’s actions are predictable rather than unpredictable (i.e. higher action similarity).

## Experiment 1

### Methods

#### Participants

Forty-eight right-handed participants (15 males; mean age 23.9; SD 3.8) took part in the experiment. An a-priori power analysis using G*Power (Faul et al., 2013) showed that a sample size of 48 provides sufficient statistical power (1 − β > 0.80, α = 0.05), to effectively induce vicarious agency (ηp2 = 0.19, Wegner et al., 2004). All participants had normal or corrected-to-normal vision. Prior to the experiment, each participant gave written informed consent to participate in the study. All the procedures were conducted in accordance with ethical guidelines of the local ethics committee of the University of Muenster and the Declaration of Helsinki. After the experiment, participants received course credits or monetary compensation (5 euro).

#### Stimuli and apparatus

**Fig. 1:**
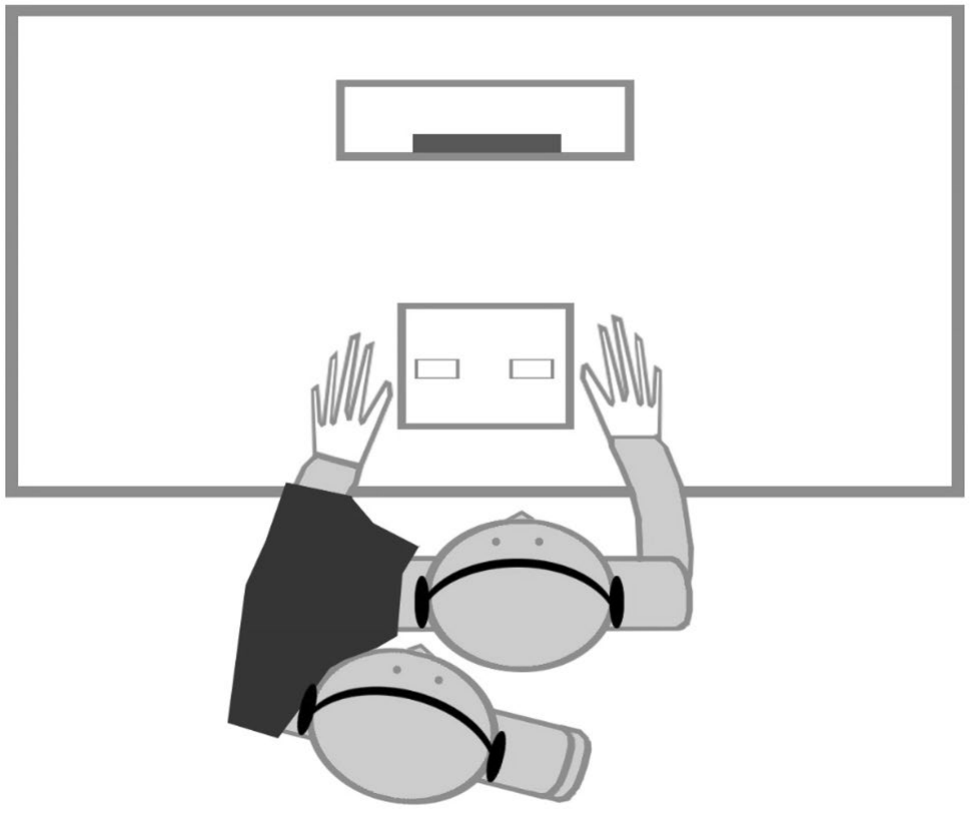
Experimental set-up. The headphones were only used to induce vicarious agency. The joint Simon task made use of visual rather than auditory stimuli

Participants were seated in a dimly lit room in front of a CRT monitor (19 inches). Participants’ head was stabilized by means of a chinrest positioned at 35 cm in front of the monitor. A same-sex confederate was seated behind the participant on the left side, such that the participant was unable to see the confederate entirely during the experiment. In order to induce in the participant a sense of agency over the confederate’s hand, a black piece of cloth was used to cover the participant’s and the confederate’s left arms, which facilitates the perception that the confederate’s left arm was part of the participant’s body. Additionally, in order to minimize the difference between the participant’s right hand and the confederate’s left hand, participant and confederate wore a white glove on the right and left hand, respectively (see Fig. 1).

Visual stimuli consisted of a white square and a white diamond (1.9° × 1.9°) displayed on a black background. Stimuli could either appear to the left or to the right side (9.5°) of a centrally presented fixation cross. As experimental software, ERTS version 3.33e was used (Beringer, 2000). Responses were given by means of two custom made response buttons positioned on a desk on the right and left side of the monitor middle line. Response buttons were kept at fixed distance of 24 cm from each other.

### Task and procedure

The experiment was subdivided in two main phases: An induction phase and a Joint Simon task phase.

### Induction phase

The induction phase started with a pure tone (400 Hz, duration: 300 ms) presented via headphones to the participant and the confederate. The sound served as a warning for an incoming verbal instruction (in German). The verbal instruction, read by a female voice, consisted of a request to perform a specific action. Verbal instructions were sent simultaneously to the participant and the confederate through headphones. Participants were allowed to perform exclusively right-hand actions while the confederate performed only left-hand actions. After hearing the instruction, participant and confederate had 7 s to perform their respective actions.

The required actions consisted of simple movements such as making an OK sign, waving or pointing toward the screen. Each action could be presented in three different versions: the right-hand version (e.g. “wave three times with your right hand”), the left-hand version (e.g. “wave three times with your left hand”) and the both hands version (e.g. “wave three times with both hands”).

Crucially, one group of participants was exposed to consistent instructions, where participant and the confederate received exactly the same instructions, and participants were always aware of the future confederate’s actions during the agency induction. For example, in the case of a left-hand consistent instruction both participant and confederate heard the instruction “wave three times with your left hand” and the confederate performed this action accordingly. Another group of participants was instead exposed to inconsistent instructions, where participants and confederate always received a different instruction, and participants were never aware of the future confederate’s actions. For example, in the case of a *left-hand* inconsistent instruction, the participant received the instruction: “wave three times with your left hand” whereas the confederate received the instruction: “point to the screen with your left hand” and performed the action accordingly, thus producing an inconsistency between the participant’s current knowledge and the actual action performed by the confederate.

The induction phase lasted approximately 3.3 min and consisted of the presentation of 26 consecutive instructions that were pseudo-randomly selected from a sample of 36 pre-recorded instructions. The stimulus presentation during the induction phase was done by means of a custom programmed Matlab (The MathWorks Inc., Natick, MA) routine.

The induction phase aimed at promoting in the participants two distinct degrees of vicarious agency over the confederate’s actions before running the joint Simon task: a low vicarious agency induction (inconsistent instructions) and a high vicarious agency induction (consistent instructions). These two types of induction were obtained by manipulating the experience of control over the confederate’s actions. In line with a study from (Wegner et al., 2004), we hypothesized that hearing consistent instructions leads to high vicarious agency, while hearing inconsistent instructions leads to low vicarious agency. The idea is that when vicarious agency during induction is high (vs. low), and participants know the confederate’s task rule in the subsequent joint Simon task, the representation of the confederate’s actions should be activated more strongly (i.e. increased self–other integration). Note that this prediction relies on the assumption that self–other integration as induced by our vicarious agency manipulation will transfer from the induction to the joint Simon task. Similar transfer of processing modes (Iani et al., 2014; Ruissen & De Bruijn, 2016) and meta-control states (Liepelt & Raab, 2021) have been demonstrated before in the joint Simon task.

### Joint Simon task phase

The setting employed in the induction phase was also kept during the Joint Simon task phase. The task consisted of a Joint go-nogo version of the Simon task (Liepelt et al., 2011; Sebanz et al., 2003), in which either a square or a diamond was randomly presented to the left or to the right side of the screen. Participants always responded to the diamond while the confederate responded to the square. Participants responded by pressing the right response button and were seated on the right side of the confederate to allow participants to represent their own actions in spatial reference to the confederate (i.e., as right vs. left). Diamonds presented on the right side of the screen would be compatible with this joint action representation, while diamonds presented on the left side of the screen would be incompatible with this joint action representation.

Each trial started with the presentation of a central fixation cross (250 ms) and was followed by the presentation of one of the two visual stimuli (150 ms) together with the fixation cross. In case of a correct response, the fixation cross was presented for 300 ms to not interrupt the processing flow. In case of a wrong response, the error feedback “Fehler” (German for error) was displayed for 300 ms. If no response was given within 1800 ms, the feedback “zu langsam” (German for too slow) was shown for 300 ms. Following the response there was a constant inter-trial interval of 1750 ms.

### General procedure

Participants were subdivided into the following two groups: a low vicarious agency and a high vicarious agency group, thus each participant was subjected to a unique type of induction phase. Beyond a general explanation of the experimental procedure, none of the subjects was informed about the goal of the experiment.

The experiment began with the Induction phase (low or high vicarious agency), followed by a brief questionnaire (see “Questionnaire” section) to assess participant’s subjective experience of agency in the induction. The questionnaire was immediately followed by the Joint Simon phase. Prior to the first experimental block, participants performed a short practice, followed by 2 blocks (128 trials) of the actual experiment. Trials were uniformly distributed between 2 conditions (compatible and incompatible). The same procedure consisting of an induction phase, a questionnaire, and two blocks of the Joint Simon task was administered twice.^1^ The experiment ended with another (i.e., third) completion of the questionnaire.

### Questionnaire

The questionnaire aimed at assessing the degree of agency experienced by each participant. The questionnaire was partially based on the questionnaire used in Wegner et al. (2004). It consisted of five distinct questions as follows:

1. To which degree could you predict the movement of your partner’s hand?
2. To which degree did you feel that you could control your partner’s arm?
3. To which degree did you feel that you could intentionally move your partner’s arm?
4. To which degree did you feel that your partner’s arm belonged to you?
5. Did your partner’s arm disturb or annoy you?

Participants were asked to answer the questions by using a scale from 1 to 7, where 1 signified “Not at all” and 7 “A lot”. The questionnaire was filled after each induction phase and immediately after the end of the experiment. Internal validity was satisfactory with an overall Cronbach’s alpha of 0.72 (α_T1_ = 0.56, α_T2_ = 0.69, α_T3_ = 0.73), which could be further enhanced by dropping the fifth item that measured the experience of agency more indirectly through downstream emotional consequences (α_overall_ = 0.79, α_T1_ = 0.69, α_T2_ = 0.79, α_T3_ = 0.77). On average, participants scored relatively low on experienced agency (*M*_overall_ = 3.0, SD = 1.1, *M*_T1_ = 2.9, SD = 1.1; *M*_T2_ = 3.0, SD = 1.3; *M*_T3_ = 3.3, SD = 1.3).

### Data analysis

For the statistical analysis, responses were considered as correct when the button press occurred within a time interval of 150–1000 ms after target onset (Liepelt et al., 2011; Röder et al., 2007). No response was classified as outlier. Correct reaction times were then subjected to a 2 × 2 mixed analysis of variance (ANOVA) with a between-subjects factor: Vicarious Agency (Low and High) and a within-subjects factor: Compatibility (Compatible and Incompatible trials).

The questionnaires were analyzed in the following way: A mean of each question across the three questionnaires (see “Task and Procedure” section) was taken in order to see the contribution of each question to the general agency assessment, then the scores were collapsed across all questions (except for question 5, whose exclusion improved Cronbach’s alpha of the questionnaire) to obtain a unique agency index for each participant of the two groups. Hence the agency indices relative to each group (low or high vicarious agency) were compared by means of a Wilcoxon rank sum test. Additionally, a Spearman correlation was performed to test any potential relationship between the agency index and a compatibility index obtained by subtracting incompatible reaction times from compatible reaction times.

Data and statistical analysis was performed using Matlab (The MathWorks Inc., Natick, MA) custom routines and the R environment (version 4.0.5 and library *psych* 2.6.1, https://CRAN.R-project.org/package=psych).

## Results

### Manipulation check

The analysis of the questionnaire revealed that overall the High vicarious agency group (*M* = 3.71, SD = 1.67) experienced a significantly higher degree of agency than the Low vicarious agency group (*M* = 2.35, SD = 1.17), *W* = 492, *p* < 0.001. A comparison of the High vs. Low vicarious agency groups on the three separate measurements of agency revealed a significant difference on the first (*M*_high_ = 3.6, SD = 0.9 vs. *M*_low_ = 2.1, SD = 0.6, *W* = 491, *p* < 0.001) and second (*M*_high_ = 3.9, SD = 1.1 vs. *M*_low_ = 1.9, SD = 0.7, *W* = 499.5, *p* < 0.001) measurements, which immediately followed the induction phases. The difference was no longer significant on the third measurement (*M*_high_ = 3.6, SD = 1.3 vs. *M*_low_ = 3.0, SD = 1.3, *W* = 362.5, *p* = 0.13), which was administered after the last two blocks of the joint Simon task.

### Main findings

**Fig. 2:**
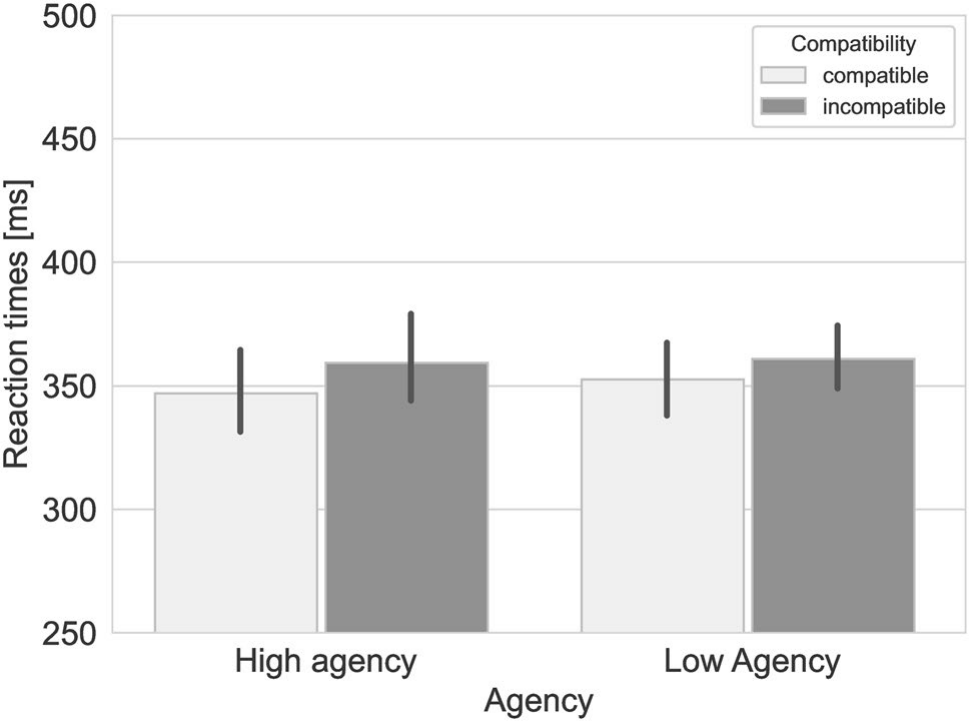
Average reaction times for compatible and incompatible trials in the high agency and low agency conditions of study 1. Error bars represent 95% confidence intervals

The mixed ANOVA revealed uniquely a main effect of Compatibility (*F*(1,46) = 20.53, *p* < 0.001; general η^2^ = 0.02) indicating that compatible responses (*M* = 350 ms, SD = 40) were generally faster than incompatible responses (*M* = 360 ms, SD = 38). No main effect of Vicarious Agency was observed (*F*(1,46) = 0.11, *p* = 0.74; general η^2^ = 0.002). Crucially, no significant interaction of Compatibility × Vicarious Agency (*F*(1,46) = 0.8, *p* = 0.4; general η^2^ = 0.0004) was observed, indicating that the compatibility effect was not modulated by Vicarious Agency. Figure 2 shows mean RT’s for each cell in the design. Errors were not further analyzed due the low occurrence (High vicarious agency: Compatible = 2.1% false alarms, 0.8% misses, Incompatible = 2.8% false alarms, 0.4% misses; Low vicarious Agency: Compatible = 2.5% false alarms, 0.0% misses, Incompatible = 2.5% false alarms, 0.0% misses).

No significant correlations were observed between the agency index and the compatibility index (*r*_overall_ = − 0.18, *p* = 0.2; *r*_T1_ = − 0.05, *p* = 0.7;, *r*_T2_ = − 0.09, *p* = 0.53;, *r*_T3_ = − 0.23, *p* = 0.1). This was the case both within the High vicarious agency group (*r*_overall_ = -0.30, *p* = 0.15) and Low vicarious agency group (*r*_overall_ = -0.17, *p* = 0.4).

## Discussion

Although the manipulation check revealed a successful induction of vicarious agency replicating the findings of Wegner et al. (2004), a general feeling of vicarious agency did not modulate the joint Simon effect. This modulation may have been absent because we manipulated (in)consistent thoughts about the co-actor’s actions prior to the joint Simon task without affecting the predictability of the co-actor’s action during the joint Simon task. In line with this notion, the manipulation check revealed that there was no longer a difference in experienced agency between the low vs. high vicarious agency groups after the completion of the joint Simon task. As such, our vicarious agency manipulation may have been too much detached from the joint Simon task, in which the co-actor’s responses were 100% predictive through the stimuli (e.g., squares always indicated that the co-actor would respond) provided that errors are not taken into account. In other words, the experience of vicarious agency may not transfer to another task setting, and in this case may in fact have been completely counteracted by action predictability during the joint Simon task. Hence, a more specific form of action predictability within the joint Simon task may be needed. In the next experiment, we therefore aimed to manipulate prior consistent thoughts about a co-actor’s action more directly in the joint Simon task.

## Experiment 2

In Experiment 2, we manipulated the prior consistent thoughts about a co-actor’s action in the joint Simon task through action prediction. The experience of agency crucially relies on such prior consistent action-related thoughts (Chambon et al., 2013; Wegner & Wheatley, 1999). Consequently, experiences of agency are typically also enhanced when actions are predictable (Haggard & Chambon, 2012; Moore & Obhi, 2012; Silver et al., 2021; van der Weiden et al., 2011b), even when it concerns other people’s actions (Bolt & Loehr, 2017; Silver et al., 2021; Wegner et al., 2004). As such, action prediction can be considered to be a strong agency cue. Note, however, that in a typical joint action task, it is rather unambiguous who performs which action (in contrast to the vicarious agency manipulation in E1), and that action prediction would therefore more likely be a cue for joint (or we-)agency.

Here we aim to build on previous research on action prediction by testing if it modulates self-other integration. We predict self–other integration to be stronger when the co-actor’s actions are predictable rather than unpredictable (i.e., higher action similarity). In contrast to our expectation for E1 that vicarious agency would increase the joint Simon effect, we expect the manipulation of action prediction to decrease the joint Simon effect in the unpredictable condition compared to the normal (baseline) situation where the co-actor’s actions are predictable.

### Methods

#### Participants and design

Ninety undergraduate students of Utrecht University participated in this second experiment. Participants performed the task in same-sex pairs (van der Weiden et al., 2016) and did not know each other. After excluding five participants for making too many errors (> 50%),^2^ the sample consisted of 85 participants (24 male; mean age 22.5; SD 4.8), divided over the unpredictable (*N* = 46) and predictable (*N* = 39) condition. An a-priori power analysis using G*Power (Faul et al., 2013) showed that a sample size of 85 provides ample statistical power (1 − β > 0.80, α = 0.05) to detect a within–between interaction with two groups and two measurements (correlation among repeated measures = 0.5, nonsphericity correction = 1), based on the effect of predictability on experienced agency (*d* = 0.46; Bolt & Loehr, 2017). Most participants were right-handed (*N* = 73; 7 were left-handed, 5 were ambidextrous). All participants had normal or corrected-to-normal vision. Prior to the experiment, each participant gave written informed consent to participate in the experiment. All the procedures were conducted in accordance with ethical guidelines of the local ethics committee of Utrecht University and the Declaration of Helsinki. After the experiment, participants received course credits or monetary compensation (6 euro).

The experiment had a 2 Predictability (unpredictable vs. predictable) × 2 Compatibility (incompatible vs. compatible) mixed design, with Predictability as between-subjects variable, and Compatibility as within-subjects variable.

#### Task and procedure

**Fig. 3:**
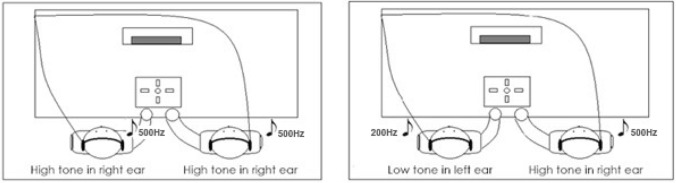
Example trials for the predictable (left) and unpredictable (right) condition

In order to establish unpredictability of the co-actor’s actions in the unpredictable condition, we had to present participants with an independent set of stimuli. In order to manipulate the predictability of the co-actor’s actions, we adapted the auditory joint Simon task of Ruys and Aarts (2010). Both participants wore headphones (Sennheiser-H201) through which they were randomly presented with low (200 Hz) or high (500 Hz) frequency tones. Participants were seated next to each other, and were informed that they would be performing a sound discrimination task in which one of them (e.g., participant seated on the left) had to respond to low tones, while the other participant had to respond to high tones. Whether the participant on the left or the right responded to low or high tones was counterbalanced between pairs. The participant on the left always responded by pressing the left key and the participant on the right always responded by pressing the right key on a response box (Cedrus® RB-530). The tones were presented to the left ear or the right ear, rendering the tones compatible (e.g., low tone presented in left ear) or incompatible (e.g., low tone presented to right ear). Figure 3 illustrates the experimental set-up.

In the predictable condition, both participants received identical stimuli (i.e., same tone in same ear), and could thus predict whether their co-actor was going to respond, or not. In the unpredictable condition, the two participants received different stimuli, such that they only heard the same tone on 50% of the trials (25% in same ear, 25% in other ear), rendering the responses of the co-actor unpredictable. Participants were not explicitly informed of whether they would hear the same tones as their interaction partner or not, but they could easily infer this from their co-actor’s actions (i.e., they sometimes both refrained from responding or responded simultaneously). Participants were also instructed not to talk to each other, which was monitored with hidden audio monitors. As a manipulation check, we asked participants to what extent they felt that the actions of their co-actor were predictable [rated on a 9-point scale ranging from 1 (not at all) to 9 (very much)].

The total trial number in both the predictable and unpredictable condition was 480. Trials were presented randomly. Prior to the first experimental block, participants performed a short practice. In between the experimental blocks, there were short 10-s breaks.^3^ Each trial started with the presentation of a tone for 250 ms, followed by a 2750-ms response window. During the whole task, a white fixation cross (+) was presented on the center of a gray colored (RGB: 96, 96, 96) computer screen.

## Results

### Data analysis

The same cut-off criteria were used as in Experiment 1. Correct reaction times were subjected to a 2 × 2 mixed analysis of variance (ANOVA) with a between subjects factor: Predictability (unpredictable vs. predictable) and a within subjects factor: Compatibility (Compatible vs. Incompatible trials). Responses below 150 ms or above 1000 ms were removed (1.28%; Ratcliff, 1993). As response cue and seating location did not interact with spatial compatibility in any of the analyses reported below (all *F*’s < 0.48), we computed mean RTs (in ms) on compatible and incompatible trials, collapsing across response cue and seating location.

### Manipulation check

Participants indicated the co-actor’s actions as more predictable in the high (*M* = 5.82, SD = 1.85) vs. low (*M* = 4.63, SD = 2.12) predictability condition, *F*(1, 83) = 7.46, *p* = 0.008, general *ɳ*^2^ = 0.01.

### Main findings

**Fig. 4:**
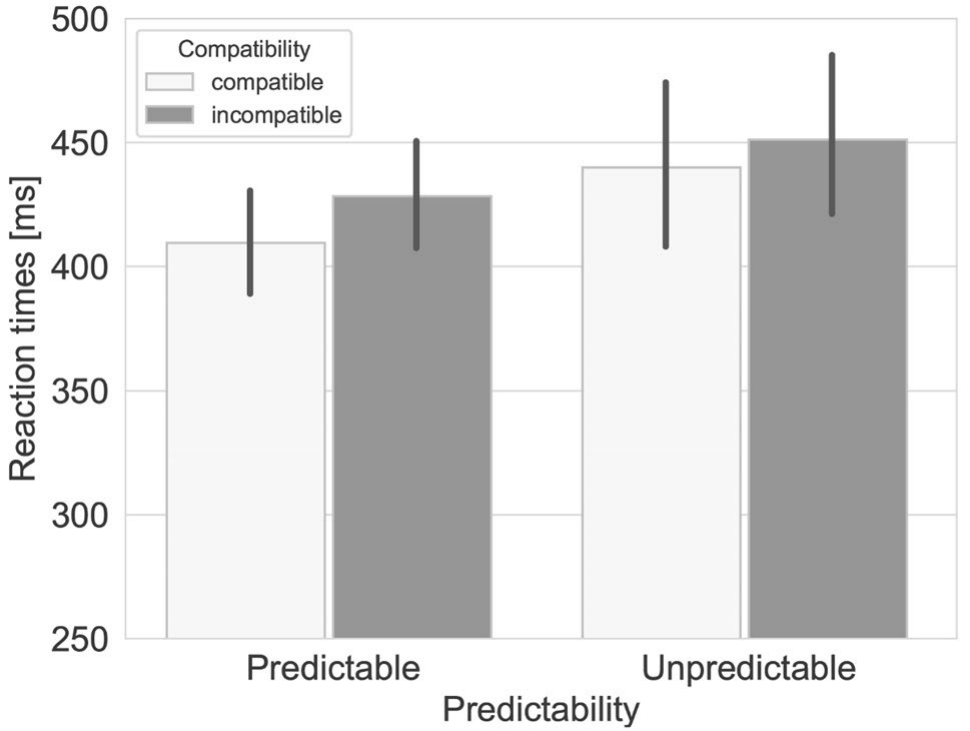
Average reaction times for compatible and incompatible trials in the predictable and unpredictable conditions of experiment 2. Error bars represent 95% confidence intervals

The analysis revealed the expected main effect of Compatibility, *F*(1,83) = 65.37, *p* < 0.001, general ɳ^2^ = 0.43. Compatible responses (*M* = 426.1, SD = 99.6) were generally faster than incompatible responses (*M* = 440.8, SD = 97.6). There was no main effect of Predictability, *F*(1,83) = 1.57, *p* = 0.21, general ɳ^2^ = 0.02. However, there was an interaction between Predictability and Compatibility (*F*(1, 83) = 3.95, *p* = 0.050, general ɳ^2^ = 0.03), such that participants’ compatibility effect was smaller when interacting with an unpredictable co-actor (*M* = 11.3 ms, SE = 2.4; *F*(1,83) = 20.25, *p* < 0.001, general ɳ^2^ = 0.13) versus a predictable co-actor (*M* = 18.7 ms, SE = 2.8; *F*(1,83) = 46.88, *p* < 0.001, general ɳ^2^ = 0.31). Figure 4 presents the mean RT’s for each cell in the design. As in Experiment 1, error rates were not further analyzed (Predictable condition: Compatible = 2.1% false alarms, 2.4% misses, Incompatible = 1.0% false alarms, 1.3% misses; Unpredictable condition: Compatible = 3.8% false alarms, 5.7% misses, Incompatible = 2.5% false alarms, 5.6% misses).

### Exploratory analyses

Our particular manipulation of predictability resulted in trials where participants responded alone, and trials where they responded simultaneously in the unpredictable condition. We had no prior expectations on whether and how this may affect participants’ actions, but we decided to explore the effects of double vs. single responses on RTs in general and on the compatibility effect. For this purpose, we first performed a 2 × 2 repeated measures ANOVA on the unpredictable condition with Number of Responses (single vs. double) and Compatibility (Compatible vs. Incompatible trials) as within-subjects factors. This analysis again revealed a main effect of Compatibility, *F*(1,45) = 22.08, *p* < 0.001, general *ɳ*^2^ = 0.19, such that compatible responses (*M* = 439.6, SE = 117.8) were faster than incompatible responses (*M* = 451.4, SE = 115.3). There was no main effect of Number of Responses, *F*(1,45) < 0.001, *p* = 0.995, general ɳ2 < ,001. However, there was an interaction between Compatibility and Number of Responses (*F*(1, 45) = 7.50, *p* = 0.009, general ɳ^2^ = 0.03), such that participants’ compatibility effect was smaller on double response trials (*M* = 6.8 ms; *F*(1,45) = 5.57, *p* = 0.02, general *ɳ*^2^ = 0.11) versus single response trials (*M* = 16.6 ms; *F*(1,45) = 26.41, *p* < 0.001, general *ɳ*^2^ = 0.37).

Next, we decided to compare the predictable vs. unpredictable condition on the effect of Compatibility for the single response trials only (as there were no double response trials in the predictable condition). A 2 × 2 mixed ANOVA again revealed the main effect of Compatibility, *F*(1,83) = 65.29, *p* < 0.001, general *ɳ*^2^ = 0.44, such that compatible responses (M = 424.6, SD = 98.6) were faster than incompatible responses (*M* = 442.1, SD = 98.5). There was neither a main effect of Predictability, *F*(1,83) = 1.55, *p* = 0.22, general ɳ2 < 0.001, nor an interaction between Compatibility and Predictability*, F*(1, 83) = 0.23, *p* = 0.63, general *ɳ*^2^ = 0.002). These findings suggest that the moderation effect of action predictability in our main analysis may be driven by lowered action interference on double response trials. But please keep in mind that the current data do not allow us to properly test whether there would be a significant three-way interaction between Compatibility, Predictability and Number of Responses.

## General discussion

In the present study, we investigated the role of vicarious agency (Experiment 1) and action prediction (Experiment 2) on self–other integration using the joint Simon paradigm. Experiment 1 showed that the manipulation of Wegner et al. (2004) that we applied to induce vicarious agency was successful. Participants experienced higher levels of vicarious agency under consistent than inconsistent conditions. However, the different levels of vicarious agency participants experienced during the induction did not modulate the strength of self–other integration in the joint Simon task. An overall joint Simon effect was present for both high and low vicarious agency conditions. This overall joint Simon effect indicates that the lack of vicarious agency did not prevent the building of a shared representation. The lack of a modulation of self–other integration through vicarious agency seems to be in line with previous findings showing that the sense of ownership over the co-actor’s hand does not increase the joint Simon effect either (Dolk et al., 2011).

In Experiment 2, we manipulated the prediction over the co-actors action in the joint Simon task. Self–other integration was weaker for the unpredictable (vs. predictable) co-actor. This can be taken as evidence that a key aspect of agency, i.e., action prediction (Haggard & Chambon, 2012) is also relevant in settings in which our actions and outcomes are related to others (Moore & Obhi, 2012; Silver et al., 2021), in this case during joint action. Hence, it appears that the joint Simon effect is not only affected by agent similarity, as it has been shown in various previous studies (Atmaca et al., 2011; Hommel et al., 2009; Stenzel et al., 2014), but also by action similarity. When a co-actor’s action is unpredictable for the actor, they perceive (a) lower agency over the co-actors actions (Bolt & Loehr, 2017; Wegner et al., 2004) and (b) show a weaker self–other integration than when the co-actor’s action is predictable. This finding also confirms the previously raised assumption that action prediction plays an important role in the joint Simon effect (Sebanz et al., 2006).

Interestingly, further exploratory analyses suggested that the effect of our action prediction manipulation could be driven by a reduced self–other integration on trials where both participants responded simultaneously (i.e., double response trials). The instructions for the joint Simon task create strong expectations concerning the task distribution, where one participant is responsible for responding to low tones, and the other participant is responsible for responding to high tones. On trials where both participants respond, the expectations regarding the task distribution are violated, thus producing a prediction error, which may lower the we-experience during joint action (Bolt et al., 2016). In addition, participants may feel that they failed to successfully discriminate the sounds when both (or neither) responded on a given trial, and people typically experience less agency over performance failures in both individual (Kip et al., 2021; Van der Weiden, Aarts, et al., 2013; Van der Weiden, Aarts, et al., 2013; van der Weiden, Ruys, et al., 2013; van der Weiden, Ruys, et al., 2013) and joint action contexts (cf. Pacherie, 2012). As prediction errors and attributions of failure would not occur on (correctly executed) single response trials, this makes them more or less comparable to a typical trial in a joint Simon task where actions are predictable. In line with this notion, the compatibility effect was indeed similar for single response trials in the predictable and unpredictable conditions. However, these exploratory findings need to be interpreted with caution and require future replication.

### Strengths and limitations

Although action unpredictability reduced the compatibility effect by 40%, statistically the effect was small in size. One reason for the small effect size could be that even though actions were either completely predictable or completely unpredictable, participants on average still perceived their co-actors’ actions as somewhat predictable in both conditions. That is, on a scale from 1 to 9, participants in the unpredictable condition rated action predictability just below the scale midpoint (*M* = 4.63), while participants in the predictable condition rated action predictability just above the scale midpoint (*M* = 5.82). This suggests that there might be room for stronger manipulations of action predictability to better determine its full potential on self–other integration in joint action.

The current study is the first to test the role of social agency in the joint Simon effect, employing two different agency inductions. As we only found an effect for action predictability, and not for vicarious agency, it is relevant to discuss several methodological and conceptual differences that may explain this divergence in findings.

First, the divergent findings seem to be consistent with the cooperation continuum of social agency proposed by Silver et al. (Silver et al., 2021), in which joint (or we-)agency (e.g., as induced by enhanced action predictability; Bolt & Loehr, 2017) is associated with the highest degree of cooperation. Yet, in contrast to the experience of joint agency and in contrast to our prediction, the experience of controlling one’s own and one’s co-actor’s actions oneself (i.e., vicarious agency) might produce the ironic effect of becoming oblivious to the presence of another actor (perceiving the situation as acting in social isolation). As such, the task in fact becomes less social and less cooperative in nature. Because the joint Simon effect has been shown to depend strongly on cooperation vs. competition (Iani et al., 2011; Liepelt & Raab, 2021; Mendl et al., 2018) and is weaker in non-social vs. social situations (Dolk et al., 2013; Müller, Brass, et al., 2011; Müller, Kühn, et al., 2011; Stenzel et al., 2012), one could argue that—due to perceived social isolation—the experience of vicarious agency would diminish the joint Simon effect compared to the low agency condition. However, the low agency condition may similarly have created social distance between the co-actors, and hence may also have diminished the joint Simon effect. Therefore, the perceived social isolation/distance may explain the absence of an interaction effect between agency and compatibility in Experiment 1.

On the other hand, if the experience of vicarious agency would indeed create a perception of fully controlling both actions oneself, one could in fact argue for a larger compatibility effect in the high (vs. low) vicarious agency condition. After all, the Simon effect is typically stronger when people have to coordinate their own (e.g., left and right hand) actions (as in the standard Simon task) compared to when they have to coordinate their actions with the actions of others (as in the joint Simon task). Hence, the absence of an interaction effect between agency and compatibility in Experiment 1 may instead suggest that participants in the high vicarious agency condition did not experience agency over their co-actor’s actions to the extent that they felt that they were fully controlling both responses themselves. This logic seems to be supported by our data, which shows that– despite a significant increase in experienced agency—participants in the high vicarious agency condition still reported feeling only moderately in control over the co-actor’s actions (i.e., 3.71 on a scale from 1 to 7), and this feeling did not seem to last throughout the joint Simon task.

Second, whereas we employed a visual Simon task in Experiment 1, we employed an auditory Simon task in Experiment 2. Although the underlying processes that give rise to the joint Simon effect are similar for both modalities (and as such would be expected to be modulated by experiences of agency in the same way), the auditory Simon task typically produces larger RT’s and compatibility effects than the visual Simon task (D’Ascenzo et al., 2018), which can also be seen in our data. Such a larger compatibility effect may offer more room for modulation effects.

Third, the two experiments differed in how close the co-actor was seated to the participant. That is, in Experiment 1, the co-actor was seated directly behind the participant (creating the illusion of being one entity), while in Experiment 2, the co-actor was seated at a small distance within arm’s length. Several studies have investigated the effect of joint action within and beyond peripersonal space (Guagnano et al., 2013; Iani et al., 2021; Welsh et al., 2013), and suggest that as long as co-actors can reach each other’s response keys, compatibility effects are likely to occur. The significant compatibility effects in both Experiment 1 and 2 support this notion, as in both Experiments the co-actor’s response key was within reach. However, it remains an open question whether and how interpersonal distance may affect the sense of agency and, as such, its modulating role in self–other integration.

Fourth, our vicarious agency manipulation was mainly aimed at enhancing the sense of agency above baseline (though inconsistent hearing instructions may also have lowered the sense of agency below baseline). Yet, as one’s co-actor’s actions in the joint Simon task are typically predictable (baseline), rendering the co-actor’s actions unpredictable would lower action predictability (and presumably joint agency) below baseline. It would make intuitive sense that lowering people’s sense of agency over the actions of their co-actors below baseline would be more disruptive for self–other integration than boosting agency would be advantageous. Although there is no direct empirical evidence to support this claim, it may partly explain why we only found an effect for our manipulation of action predictability and not for vicarious agency.

Finally, and perhaps most importantly, as already addressed in the discussion of Experiment 1, our results suggest that unlike transfer of processing modes and meta-control states (Iani et al., 2014; Liepelt & Raab, 2021; Ruissen & De Bruijn, 2016), self–other integration in the form of vicarious agency does not necessarily transfer to another task. Because the results of Experiment 2 indicate that action predictability modulates the joint Simon effect, it is likely that the full predictability of the co-actor’s responses in Experiment 1 countered our vicarious agency induction, which also relied on action predictability. As such, it may be required to maintain the (vicarious) agency induction during the joint Simon task, as in the induction of action prediction in Experiment﻿ 2. In fact, employing the experimental set-up of Experiment 1 (with the co-actor’s arm psychologically replacing the participant’s arm) could further strengthen the effects of action prediction as observed in Experiment 2, where the co-actor’s actions were completely predictable (vs. completely unpredictable) throughout the joint Simon task.

### Implications

The current findings do not only extend previous research on agent similarity in joint action, but also contribute to our knowledge of the implications of experiencing agency in social interaction. Most research on agency experiences has focused on how these experiences emerge, while only few so far have demonstrated social implications, e.g. for feelings of responsibility (Cracco et al., 2016; Moretto et al., 2011) and empathy (Caspar et al., 2020; Lepron et al., 2014). The current findings indicate that a key feature of the sense of agency — namely action prediction — can also contribute to the coordination of our actions with other people.

### Future directions

Whether an effective modulation of self–other integration requires a conscious experience of agency cannot be answered with the present study. In principle action predictability could also have induced an implicit sense of agency outside of conscious awareness. An interesting direction for future research would be to test whether and how (online) implicit and explicit experiences of agency contribute to the joint Simon effect.

Based on our findings another interesting direction for future research would be to test how different aspects of the agency experience contribute to joint action representations. For example, it has been suggested that actions that are perceived to be effortful (rather than effortless) are accompanied by stronger experiences of agency. In other words, people feel more agentically involved when they have to work hard for their goals (see also Preston & Wegner, 2009). In contrast, a degree of effortlessness can give the impression of events happening to a person instead of being caused by that person (Csikszentmihalyi et al., 2005). In line with this notion, enhanced perceptions of effort caused by squeezing a handgrip (Preston & Wegner, 2007), pulling stretch bands (Demanet et al., 2013), or using one’s non-dominant hand (Damen et al., 2014) have indeed been shown to increase the sense of agency, even if the effort is unrelated to the action over which agency is assessed. Hence, if one would experience effort during one’s co-actor’s action performance, one might represent these actions more strongly.

Another, perhaps seemingly contradictory agency cue is the fluency of action selection. Note, however, that action selection fluency and effortful action execution are independent from each other. That is, even when action selection may be fluent, the subsequent performance and regulation of the selected action can still be quite effortful (e.g., working out, preparing a fresh meal, or abiding by social norms). For example, it may be a no-brainer to go and help a friend who is clearly struggling to move a heavy table, yet helping to carry the table may be quite effortful. Ofte﻿n, people can choose from a range of possible (and perhaps conflicting) actions. Hence, action selection can be quite burdensome. In the joint Simon task, action co-representation may result in such action selection conflict due to stimulus–response incompatibility. In such cases, experienced agency may be reduced, as research has shown that experienced agency over self-produced actions is typically strongest when action selection is smooth (e.g., on compatible compared with incompatible Stroop trials; Morsella et al., 2009; Sidarus & Haggard, 2016; Sidarus et al., 2017; Wang et al., 2017; Wenke et al., 2010). Intriguingly, this effect of action fluency on experienced self-agency even occurs when action predictions are externally induced (e.g., stimulus-driven) and originate from outside our motor control system (Chambon & Haggard, 2012; Martiny-Huenger et al., 2015; Sidarus & Haggard, 2016; Wegner et al., 2004; Wenke et al., 2010). Although action selection fluency as a consequence of increased action predictability (e.g., by subliminally priming the stimuli prior to their actual presentation, predicting who will need to respond) could enhance experienced agency during the joint Simon task, it is also likely to debilitate the functionality of the Simon task as it may counteract any response interference as a consequence of self–other integration. As such, other self–other integration measures that do not rely on response interference (e.g., Inclusion of Other in the Self Scale; Aron et al., 1992) may be needed to measure the effect of action selection fluency.

Another valuable avenue for future research would be to disentangle effects of action predictability and simultaneous responding. In our manipulation, action predictability implied that participants would sometimes act simultaneously in the unpredictable condition, which did not occur in the predictable condition. As such, we were unable to test a potential three-way interaction between compatibility, predictability, and simultaneous responses. To solve this issue, future research could add simultaneous response trials to the predictable condition as well, where these simultaneous responses would then be predictably cued (e.g., by a double tone of the same total duration as the single tones).

Additionally, researchers could look into potential moderators of such task distribution violation effects. In daily life it also sometimes happens that despite a prior agreement on the distribution of certain tasks, the other person unexpectedly takes over one’s responsibility, disrupting the flow of action coordination. However, depending on the nature of the task, the act of taking over someone else’s task can be either egotistic or pro-social, and may differently impact self–other integration. For example, unexpectedly doing the dishes while one’s partner was supposed to do them is a welcome violation of task distribution and can be perceived as highly cooperative. As such, it may enhance self–other integration. In contrast, if someone raises your excellent idea in a meeting and takes credit for it while you both agreed that you would bring it up, this is very frustrating and may decrease self–other integration. Also higher level interpretations of why someone is taking over your task (e.g., out of kindness vs. because they deem you incompetent) may influence the effect of task distribution violations on self–other integration.

Other manipulations of action predictability that do not result in double response trials and the violation of task distribution are also worth investigating. Research on the experience of agency over self-produced actions has shown that time delays between action and outcome lowers the sense of agency (Wen, 2019) and may thus also lower the sense of joint agency during action coordination. In the joint Simon task, the timing of the co-actor’s actions could be rendered unpredictable by presenting the stimuli to the two participants at different points in time or by omitting some of the stimuli for one of the participants. As a consequence, one’s co-actor may unexpectedly respond when one has not (yet) heard a tone. Also, one’s co-actor may respond unexpectedly late or not at all. A prediction error in this case would not arise because of a violation of task distribution, but because of violations of the task rule to respond as quickly as possible.

Furthermore, it is important to consider different levels of action predictability. In the current line of research we only created conditions of completely predictable or completely unpredictable actions. Yet, different patterns may emerge when rendering the co-actors’ actions just slightly unpredictable. For example, in one study (Klempova & Liepelt, 2016), the co-actor responded – against the expectation – simultaneously with the participant in 7% of the trials. Results showed that after such unexpected trials, the joint Simon effect temporarily increased. A possible explanation for this increased joint Simon effect is that the unexpected action increases the attention to the co-actor’s action—perhaps even with the aim of re-establishing one’s sense of agency—resulting in a larger joint Simon effect on subsequent predictable trials (Dolk et al., 2013; Hommel, 1993). However, when one fails to predict one’s co-actor’ actions in the majority of the time, one no longer experiences agency over these actions, resulting in reduced joint action representations. It would be interesting to test under which levels of predictability the joint Simon effect starts to (temporarily) increase or decrease.

It is also noteworthy that people typically tend to make themselves predictable during joint actions with the aim to enhance action coordination. In a study that demonstrated this strategic use of action predictability, participants had to react to a certain stimulus at the exact same time as their co-actor (Vesper et al., 2011, 2013, 2016). To coordinate their actions in time, they reduced their reaction time variability by reacting as soon as possible. This strategy was not used in the mere presence of another actor when no action coordination was required. It may well be the case that such reduced response variability also enhances agency experiences, which may mediate the effect on joint action coordination. However, this argumentation is speculative and awaits future testing.

Finally, it would be interesting to see how the joint Simon effect relates to the standard Simon effect under conditions of vicarious agency and action prediction. By including a standard Simon task version in the study design, or by testing response features (e.g. visual, auditory) that are unique to the standard Simon effect, one may determine whether vicarious agency actually leads people to represent the co-actor’s action as their own (and would basically turn the joint Simon task into a standard Simon task).

## Concluding remarks

Joint action representations are essential for action coordination. Therefore, it is important to understand when people feel in control over another person’s actions and how this social agency impacts self–other integration (Silver et al., 2021). Previous research has convincingly demonstrated that the extent to which the co-actor is perceived as agentic is positively related to the joint representation of actions. Extending this line of research, we presented novel evidence for the crucial role of action prediction in joint action. In doing so, we hope that the present research may offer new directions in the study of joint action, to further our understanding of the conditions that facilitate smooth action coordination.

## Footnotes

1 There was a main effect of repetition, such that RT’s were higher in the first (*M* = 357) compared with the second (*M* = 352.3) joint Simon task, *F*(1, 46) = 5.4, *p* = 0.02, *eta sq* = 0.004. But since there were no interaction effects with compatibility or agency (all *p*’s > 0.36), we collapsed the data over the two repetitions of the joint Simon task.

2 Two participants seemed to have mixed up the instructions (they pressed when they shouldn’t and vice versa; ≥ 210 false hits and ≤ 238 misses). The other 3 participants responded on only 89–123 trials of the 240 trials on which they should have responded, while accurately refraining from responding on trials where they should not have responded (their number of false hits ranged from 1 to 5).

3 The breaks were kept short to limit the likelihood that participants would commence a conversation about the experiment.
